# Data‐enabled predictive control for quadcopters

**DOI:** 10.1002/rnc.5686

**Published:** 2021-07-13

**Authors:** Ezzat Elokda, Jeremy Coulson, Paul N. Beuchat, John Lygeros, Florian Dörfler

**Affiliations:** ^1^ Automatic Control Lab (IfA) ETH Zürich Zürich Switzerland

**Keywords:** data‐driven control, predictive control, quadcopters

## Abstract

We study the application of a data‐enabled predictive control (DeePC) algorithm for position control of real‐world nano‐quadcopters. The DeePC algorithm is a finite‐horizon, optimal control method that uses input/output measurements from the system to predict future trajectories without the need for system identification or state estimation. The algorithm predicts future trajectories of the quadcopter by linearly combining previously measured trajectories (motion primitives). We illustrate the necessity of a regularized variant of the DeePC algorithm to handle the nonlinear nature of the real‐world quadcopter dynamics with noisy measurements. Simulation‐based analysis is used to gain insights into the effects of regularization, and experimental results validate that these insights carry over to the real‐world quadcopter. Moreover, we demonstrate the reliability of the DeePC algorithm by collecting a new set of input/output measurements for every real‐world experiment performed. The performance of the DeePC algorithm is compared to Model Predictive Control based on a first‐principles model of the quadcopter. The results are demonstrated with a video of successful trajectory tracking of the real‐world quadcopter.

## 1 INTRODUCTION

The analysis and design of control systems is traditionally addressed using a model‐based control approach where a model for the system is first identified from data, and the control policy is then designed based on the identified model. The system identification step is often the most time‐consuming and challenging part of model‐based control approaches.^1^, ^2^ System identification often requires expert knowledge and partial system models,^3^ and unless the control objective is taken into account during the identification process, the obtained model may not be useful for control.^4^ These observations as well as the advancements in sensing and computation technologies have motivated a tendency toward data‐driven control methods yielding many successes.^5^, ^6^, ^7^, ^8^ Such methods bypass the traditional model‐based control approach, and design control inputs directly from data. These so‐called direct data‐driven methods for control design benefit from ease of implementation on complex systems where system identification is too time‐consuming and cumbersome. Among these data‐driven methods are learning‐based and adaptive Model Predictive Control (MPC) approaches, where the unknown system dynamics are substituted with a learned model which maps inputs to output predictions.^9^, ^10^, ^11^, ^12^, ^13^ However, such methods still require learning an input/output model and often involve (stochastic) function approximation by means of neural networks or Gaussian processes, which come with their own tuning challenges and can be inconsistent across applications.^14^

One algorithm that does not require any function learning or system identification is the so‐called data‐enabled predictive control (DeePC) algorithm.^15^ Instead, this algorithm *directly* uses previously measured input/output data to predict future trajectories. The previously measured input/output data from the system act as *motion primitives* that serve as a basis for the subspace of possible system trajectories. The DeePC algorithm builds on the seminal work on linear time invariant (LTI) systems by Willems et al., specifically what is known as the *fundamental lemma* in behavioral systems theory.^16^ This result was used by Markovsky et al. for the first time for control purposes allowing for the synthesis of data‐driven open loop control for LTI systems.^17^ The DeePC algorithm extended this method to closed‐loop control and was implemented in a receding horizon optimal control setup. This algorithm was shown to be equivalent to MPC for deterministic LTI systems,^15^ and was later extended giving guarantees on recursive feasibility and closed‐loop stability.^18^ Additionally, numerical case studies have illustrated that the algorithm performs robustly on some stochastic and nonlinear systems and often outperforms system identification followed by conventional MPC.^19^, ^20^, ^21^

Several other data‐driven control methods have been proposed that make use of input/output data in similar ways as DeePC. One method uses the fundamental lemma to synthesize stabilizing output feedback controllers solving the linear quadratic regulation problem using only input/output data.^22^ Other methods use previously measured input/output trajectories as motion primitives to compute minimum energy inputs,^23^ or produce new control inputs for LTI systems.^24^ All of these methods, including the DeePC algorithm, rely on the linearity property. For nonlinear systems, data‐driven control methods that make use of motion primitives to synthesize new trajectories have been proposed.^25^, ^26^ Common to these methods is a nonlinear data‐fitting step in the generation of the motion primitives. One approach uses sparse identification to fit the raw data to a predefined library of nonlinear primitives.^25^ An approach tailored to robotics applications learns motion primitives from demonstration trajectories by estimating the parameters of nonlinear Gaussian basis functions.^26^ By contrast, DeePC directly uses raw data sequences as motion primitives, and there is no data‐fitting step. It relies on a robustifying regularization, which is incorporated directly in the optimal control objective, to address nonlinearity and stochasticity.^15^, ^19^, ^27^

The focus of this paper is on implementing this robustified, regularized variant of the DeePC algorithm for the first time on a real‐world system. In particular, we seek to analyze how the algorithm can be applied for real‐time control of a quadcopter whose dynamics are nonlinear and the measurements are corrupted by noise. The quadcopter is a common benchmark system for verifying data‐driven control methods.^7^, ^8^, ^28^, ^29^, ^30^ It makes for an interesting benchmark because it is nonlinear, open‐loop unstable and has fast dynamics. Our main aim is to gain valuable insights on this benchmark which will assist in the implementation of our novel data‐driven control method across multiple systems.

**Contributions:** The DeePC algorithm is implemented for the first time on a real‐world system bridging the gap from theory to application. Through this, we gain key insights into choices of the algorithm's hyperparameters, providing tuning guidelines. We demonstrate that the DeePC algorithm is computationally tractable and suitable for real‐time control. A video of the DeePC algorithm performing figure 8 trajectory tracking on the real‐world quadcopter is provided here: https://doi.org/10.3929/ethz‐b‐000493419.

**Outline:** The real‐world quadcopter system, problem statement, and DeePC algorithm are introduced in Section 2. The main contributions appear in Section 3, where we present simulation analysis and experimental results, as well as a video of successful trajectory tracking of the quadcopter. We conclude in Section 4 stating some future directions of research.

## 2 SETTING

We first present the quadcopter system in Section 2.1, providing details about its input/output channels, and the first‐principles modeling that is used for simulation‐based analysis. We then formally state in Section 2.2 the quadcopter control goal as a general finite‐horizon, discrete‐time, optimal control problem. Section 2.3 recalls the DeePC algorithm, showing how it can be used to address both LTI and nonlinear stochastic control problems in a data‐driven way.

### 2.1 Quadcopter

For the purpose of simulation, we use a nonlinear, continuous‐time quadcopter model. Full details of the model derivation are provided in other works.^31^, ^32^ Here we highlight the key definitions, equations, and control architecture. The model presented is also the starting point for the model‐based control methods that are used for comparison in Section 3.4.

**FIGURE 1:**
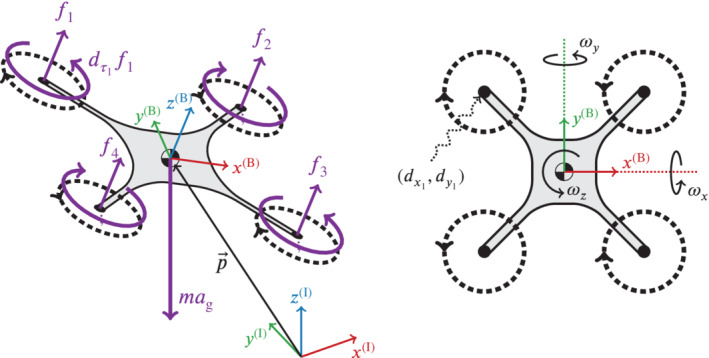
Perspective view (left) and top view (right) of the quadcopter model used for simulation; the annotations are defined in Section 2.1. The (red, green, blue) arrows represent the *inertial* and *body* frames of reference, the dashed black circles indicate the direction of rotation of the propellers, and the purple arrows show the forces and torques acting on the quadcopter model.

We define the model in terms of an *inertial* frame of reference, denoted (I), and a *body* frame of reference attached to the quadcopter, denoted (B), with the origin of frame (B) fixed at the quadcopter's center‐of‐gravity. The position of the body frame with respect to the inertial frame is denoted by $\vec{p}=\left(p_{x},p_{y},p_{z}\right)$. We use Euler angles to describe the orientation of the body frame relative to the inertial frame, and following the ZYX intrinsic Euler angle convention, we denote the roll, pitch, and yaw angles by $\vec{\psi }=\left(\gamma ,\beta ,\alpha \right)$ respectively. The angular rates about the body frame axes are denoted by $\vec{\omega }=\left(\omega_{x},\omega_{y},\omega_{z}\right)$. Thus, the model has 12 states, $\left(\vec{p},\dot{\vec{p}},\vec{\psi },\vec{\omega }\right)$, and the inputs to the model are the thrust force from each propeller, denoted $f_{i}$, i=1,…,4. The parameters required for the quadcopter model are the mass m, the mass moment of inertia J, the body frame coordinates for the center‐of‐thrust of each propeller $\left(d_{x_{i}},d_{y_{i}}\right)$, and the constant of proportionality $d_{\tau_{i}}$ that approximates a linear relation between the torque due to propeller drag and the thrust force $f_{i}$. Figure 1 visualizes this definition of the quadcopter. The nonlinear, continuous‐time equations of motion are readily derived as

(1a) $$\begin{array}{ll} \ddot{\vec{p}}= & \;\frac{1}{m}\;\overset{4}{\underset{i=1}{\sum }}\;\;f_{i}\;\left(\begin{array}{c} \cos(\alpha )\sin(\beta )\cos(\gamma )+\sin(\alpha )\sin(\gamma )\\ \sin(\alpha )\sin(\beta )\cos(\gamma )-\cos(\alpha )\sin(\gamma )\\ \cos(\beta )\cos(\gamma ) \end{array}\right)\;-\;\left(\begin{array}{c} 0\\ 0\\ a_{g} \end{array}\right), \end{array}$$

(1b) $$\begin{array}{ll} \dot{\vec{\omega }}= & \;J^{-1}\;\left(\;\left(\begin{array}{c} \sum_{i=1}^{4}\;f_{i}d_{y_{i}}\\ \sum_{i=1}^{4}\;-f_{i}d_{x_{i}}\\ \sum_{i=1}^{4}\;f_{i}d_{\tau_{i}} \end{array}\right)\;-\;\vec{\omega }\times J\vec{\omega }\;\right), \end{array}$$

where $a_{g}$ is the acceleration due to gravity. An important feature of these equations is that the equilibrium inputs are the same at all positions $\vec{p}$ and at all yaw angles α.

Most off‐the‐shelf quadcopters are equipped with an on‐board controller that allows the user to specify references instead of directly specifying the thrust force for each propeller, we refer to this as the *inner controller*. Often the manufacturer does not provide details of the inner controller and does not allow the user to bypass it. We consider a quadcopter with an inner controller that uses the data from the onboard inertial measurement unit (IMU) to track user provided references for the angular rate about the $x^{\left(B\right)}$ and $y^{\left(B\right)}$ axes of the body frame and maintains a constant yaw angle. We leave the inner controller as implemented by the manufacturer, and consider the following three inputs to the system:

- the body rate references about the $x^{\left(B\right)}$ and $y^{\left(B\right)}$ axes, denoted by $\omega_{\text{ref},x}$ and $\omega_{\text{ref},y}$ respectively, and
- the total thrust force from the propellers combined, denoted by $f_{\text{tot}}$.

**FIGURE 2:**
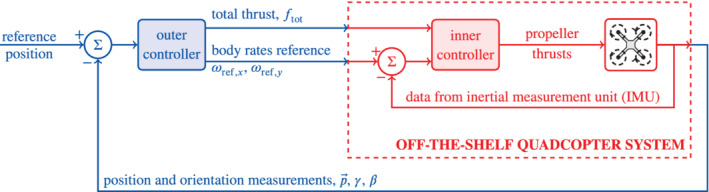
Block diagram of the cascaded control architecture used for the simulations and experiments. In an off‐the‐shelf quadcopter system, the *inner controller* is typically already implemented. Here we focus on the synthesis of the *outer controller*.

The *outer controller* adjusts these three inputs to ensure that the quadcopter tracks a position reference provided by the user, based on feedback of position and orientation measurements, $\vec{p}$, γ, and β, provided by an external motion capture system.^33^, ^34^ Our aim is to design a data‐driven outer controller for this 3 input, 5 output off‐the‐shelf quadcopter system (see Figure 2 for a schematic of the architecture). Previous work has demonstrated that it is possible to design in simulation a data‐driven controller from position and orientation measurements to propeller thrusts directly.^15^ As the goal of this work is to control a real‐world quadcopter, we consider that the inner controller on the off‐the‐shelf quadcopter constitutes a part of the *black‐box system* under investigation.

### 2.2 Problem statement

Let us consider a discretized version of the quadcopter dynamics (1), which we denote by

(2) $$\begin{array}{ll} x(t+1) & =f(x(t),u(t)),\\ y(t) & =h(x(t),u(t)), \end{array}$$

where $x(t)\in {\mathbb{R}}^{n}$, $u(t)\in {\mathbb{R}}^{m}$, and $y(t)\in {\mathbb{R}}^{p}$ are, respectively, the state, control input, and output at time $t\in {\mathbb{Z}}_{\geq 0}$. Note that even though the continuous‐time dynamics (1) are known, an analytic expression does not exist for the nonlinear discretized dynamics described by mappings $f:{\mathbb{R}}^{n}\times {\mathbb{R}}^{m}\to {\mathbb{R}}^{n}$ and $h:{\mathbb{R}}^{n}\times {\mathbb{R}}^{m}\to {\mathbb{R}}^{p}$ in (2). We purposefully abstract notation above to highlight the fact that the problem statement is not unique to a quadcopter, but can be applied to many systems with nonlinear dynamics whose linearization about the operating point is a controllable and observable LTI system (see Section 2.3). For the quadcopter, we have that, $u(t)=(f_{\text{tot}},\omega_{\text{ref},x},\omega_{\text{ref},y})\in {\mathbb{R}}^{3}$, and $y(t)=(p_{x},p_{y},p_{z},\gamma ,\beta )\in {\mathbb{R}}^{5}$. The state x(t) includes the quadcopter position, velocity, Euler angles, angular velocities, motor currents, and the states of the inner controller. From these quantities, u(t) and y(t) are what we have available for controller synthesis, while the state x(t) is regarded as unknown.

The problem of constrained finite‐horizon optimal control is considered. Given the current time $t\in {\mathbb{Z}}_{\geq 0}$, a time horizon $T_{f}\in {\mathbb{Z}}_{\geq 0}$, input and output constraint sets $𝒰\subseteq {\mathbb{R}}^{m}$, $𝒴\subseteq {\mathbb{R}}^{p}$, the goal is to design a sequence of admissible control inputs ${\left\{u(t+i)\right\}}_{i=0}^{T_{f}-1}\subset 𝒰$ such that when applied to system (2), the resulting outputs ${\left\{y(t+i)\right\}}_{t=0}^{T_{f}-1}\subset {\mathbb{R}}^{p}$ lie in the constraint set 𝒴 and minimize the stage costs given by cost function $c:{\mathbb{R}}^{p}\times {\mathbb{R}}^{m}\to {\mathbb{R}}_{\geq 0}$. More formally, we wish to solve the following optimization problem:

(3) $$\begin{array}{ll} \underset{u,y}{\text{minimize}}\; & \overset{T_{f}-1}{\underset{i=0}{\sum }}\;c(y(t+i),u(t+i))\\ \text{subject to}\; & x(t+i+1)=f(x(t+i),u(t+i)),\;\forall i\in \{0,\ldots ,T_{f}-1\}\\ & y(t+i)=h(x(t+i),u(t+i)),\;\forall i\in \{0,\ldots ,T_{f}-1\}\\ & u(t+i)\in 𝒰,\;y(t+i)\in 𝒴,\;\forall i\in \{0,\ldots ,T_{f}-1\}\\ & x(t)=\hat{x}(t), \end{array}$$

where $\hat{x}(t)$ is an estimate of the state at time *t*, typically computed by filtering the sequence of past inputs and outputs. Problem (3) is solved in a receding horizon fashion and is widely known as output‐feedback MPC. The cost function *c* can be designed by the user to attain various control objectives (e.g., regulation or trajectory tracking).

Without knowledge of system (2), solving problem (3) is no longer possible as we are unable to predict forward trajectories of the system, and estimate the current state x(t). To resolve these issues, we approach the problem in a data‐driven manner. In particular, we use the DeePC algorithm,^15^ which replaces the constraints requiring system knowledge by raw input/output data to solve an optimization problem similar to (3), and, under assumptions to be recalled next, directly equivalent to (3).

### 2.3 Data‐enabled predictive control

#### 2.3.1 DeePC for deterministic LTI systems

The DeePC algorithm has been shown to be an equivalent data‐driven method for solving (3) when the unknown system (2) is a deterministic LTI minimal realization, that is, when the dynamics in (2) are of the form

(4) $$\begin{array}{ll} & x(t+1)=Ax(t)+Bu(t),\\ & y(t)=Cx(t)+Du(t), \end{array}$$

where A,B,C,D are matrices of appropriate dimensions. Note that (4) being a minimal realization implies controllability and observability properties of the system. Several modifications have also been proposed for robustifying the algorithm against stochastic disturbances.^19^, ^27^ We first introduce the necessary preliminaries, then recall the DeePC algorithm as applied to LTI systems of the form (4), followed by the robustifying regularizations that allows the algorithm's adaptation for the nonlinear quadcopter system (2) with noisy measurements.

Let the Hankel operator which maps a sequence of signals $u={\left\{u(i)\right\}}_{i=1}^{T}\subset {\mathbb{R}}^{m}$ to a Hankel matrix * with $L\in {\mathbb{Z}}_{>0}$ block rows be denoted by

$$H_{L}(u)\;:=\left(\begin{array}{cccc} u(1) & u(2) & \ldots & u(T-L+1)\\ u(2) & u(3) & \ldots & u(T-L+2)\\ \vdots & \vdots & \ddots & \vdots \\ u(L) & u(L+1) & \ldots & u(T) \end{array}\right).$$

Definition 1 (Persistency of Excitation^16^) Let $L\in {\mathbb{Z}}_{>0}$. The sequence of signals $u={\left\{u(i)\right\}}_{i=1}^{T}\subset {\mathbb{R}}^{m}$ is called *persistently exciting of order L* if the Hankel matrix $H_{L}(u)$ has full row rank.

Note that the property of being persistently exciting of order *L* requires the length of the sequence of signals be large enough; in particular, the length must be such that T≥(m+1)L−1. Intuitively, a persistently exciting sequence of signals must be *sufficiently long* and *sufficiently rich* to excite all aspects of the dynamics (4). The DeePC algorithm relies on the following fundamental result.

Theorem 1 (Theorem 1 of Reference 16) *Let* $T_{d},L\in {\mathbb{Z}}_{>0}$ *. Let* $(u_{d},y_{d})={\left\{(u_{d}(i),y_{d}(i))\right\}}_{i=1}^{T_{d}}$ *be a trajectory of (* *4* *) of length* $T_{d}$ *such that* ${\left\{u_{d}(i)\right\}}_{i=1}^{T_{d}}$ *is persistently exciting of order* L+n *. Then* $(u,y)={\left\{(u(i),y(i))\right\}}_{i=1}^{L}$ *is a trajectory of (* *4* *) if and only if there exists* $g\in {\mathbb{R}}^{T_{d}-L+1}$ *such that*

$$\left(\begin{array}{c} H_{L}(u_{d})\\ H_{L}(y_{d}) \end{array}\right)g=\left(\begin{array}{c} u\\ y \end{array}\right).$$

The result above states that the subspace spanned by the columns of the Hankel matrix $\left(\begin{array}{c} H_{L}(u_{d})\\ H_{L}(y_{d}) \end{array}\right)$ corresponds exactly to the subspace of possible trajectories of (4). Hence, the Hankel matrix may serve as a nonparametric model for (4), one that is simply constructed from raw time‐series data and does not require any learning.

In what follows, we will see how the above theorem allows us to perform implicit state estimation as well as predict forward trajectories of the unknown system allowing us to solve an optimization problem equivalent to (3) when the system is of the form (4).

**Data collection:** Let $T_{d},T_{\text{ini}}\in {\mathbb{Z}}_{>0}$ be the length of data collection and the time horizon used for initial condition estimation, respectively. Suppose $(u_{d},y_{d})={\left\{(u_{d}(i),y_{d}(i))\right\}}_{i=1}^{T_{d}}$ is a sequence of input/output measurements collected from (4) during an offline procedure. Suppose further that the input ${\left\{u_{d}(i)\right\}}_{i=1}^{T_{d}}$ is persistently exciting of order $T_{\text{ini}}+T_{f}+n$. We partition the input/output measurements into Hankel matrices

(5) $$\left(\begin{array}{c} U_{p}\\ U_{f} \end{array}\right)\;:={\;H}_{T_{\text{ini}}+T_{f}}(u_{d}),\;\left(\begin{array}{c} Y_{p}\\ Y_{f} \end{array}\right)\;:={\;H}_{T_{\text{ini}}+T_{f}}(y_{d}),$$

where $U_{p}$ consists of the first $T_{\text{ini}}$ block rows of $H_{T_{\text{ini}}+T_{f}}(u_{d})$ and $U_{f}$ consists of the last $T_{f}$ block rows of $H_{T_{\text{ini}}+T_{f}}(u_{d})$ (similarly for $Y_{p}$ and $Y_{f}$). The data in $U_{p}$ and $Y_{p}$ will be used in conjunction with *past* data to perform implicit initial condition estimation, and the data in $U_{f}$ and $Y_{f}$ will be used to predict *future* trajectories.

**Data‐driven control and estimation:** Let $(u_{\text{ini}},y_{\text{ini}})={\left\{(u_{\text{ini}}(t+i),y_{\text{ini}}(t+i))\right\}}_{i=-T_{\text{ini}}}^{-1}$ be the $T_{\text{ini}}$ most recent past input/output measurements from the system. By Theorem 1, $(u,y)={\left\{u(t+i),y(t+i)\right\}}_{i=0}^{T_{f}-1}$ is a possible future trajectory of (4) if and only if there exists $g\in {\mathbb{R}}^{T_{d}-T_{\text{ini}}-T_{f}+1}$ satisfying

(6) $$\left(\begin{array}{c} U_{p}\\ Y_{p}\\ U_{f}\\ Y_{f} \end{array}\right)g=\left(\begin{array}{c} u_{\text{ini}}\\ y_{\text{ini}}\\ u\\ y \end{array}\right).$$

Every column of the Hankel matrix is a trajectory of the system (motion primitive), and any new trajectory (right‐hand side of (6)) can be synthesized by a linear combination of these motion primitives. Hence, given an input sequence *u* to be applied to the system, one can solve the first three block equations of (6) for *g*, and the corresponding output sequence is given by $y=Y_{f}g$. The top two block equations in (6) are used to implicitly fix the initial condition from which the future trajectory departs. To uniquely fix the initial condition from which the future trajectory departs, one must set $T_{\text{ini}}\geq \ell$, where ℓ is the lag of the system (i.e., the number of past measurements required to uniquely identify the current state of the system through back‐propagation of the dynamics (4)). This in turn implies that the predicted trajectory given by $y=Y_{f}g$ is unique.^17^ Note that the lag ℓ of the system is a priori unknown, but is upper bounded by *n*. Hence, knowing an upper bound on the state dimension *n* of the system is sufficient to obtain unique predictions.

The Hankel matrix in (6) simultaneously performs state estimation and prediction, and can thus be used as a predictive model for system (4). Substituting (6) for the unknown dynamics (4) in the optimization problem (3) gives rise to the following data‐driven optimization problem allowing for the computation of optimal control inputs without knowledge of a system model:

(7) $$\begin{array}{ll} \underset{u,y,g}{\text{minimize}}\; & \overset{T_{f}-1}{\underset{i=0}{\sum }}\;c(y(t+i),u(t+i))\\ \text{subject to}\; & \left(\begin{array}{c} U_{p}\\ Y_{p}\\ U_{f}\\ Y_{f} \end{array}\right)g=\left(\begin{array}{c} u_{\text{ini}}\\ y_{\text{ini}}\\ u\\ y \end{array}\right)\\ & u\in {𝒰}^{T_{f}},\;y\in {𝒴}^{T_{f}}, \end{array}$$

where ${𝒰}^{T_{f}}$ is the $T_{f}$‐fold cartesian product of 𝒰 (similarly for ${𝒴}^{T_{f}}$). The optimization problem (7) was shown to be equivalent to the MPC problem given in (3) when the unknown system is of the form (4).^15^ Note that the optimization problem (7) does not include any parameters that need to be estimated from data. The Hankel matrix directly uses raw data without further processing, the cost function *c* is specified by the practitioner, and the optimization variable *g* is solved for in every online iteration of the algorithm. There is no separate model‐fitting or denoising step.

#### 2.3.2 Regularized DeePC for nonlinear noisy systems

The goal of this paper is to implement the above DeePC optimization problem to control a real‐world quadcopter described above in Section 2.1. As the quadcopter dynamics do not satisfy the deterministic LTI assumption necessary to show the equivalence of the MPC optimization problem (3) and the DeePC optimization problem (7), regularizations are needed. Indeed, when the input/output data used for the Hankel matrix in (7) is obtained from a nonlinear system or is corrupted by process or measurement noise (as is the case with any real‐world application) the subspace spanned by the columns of the Hankel matrix no longer coincides with the subspace of possible trajectories of the system. In fact, in any real‐world problem setting the Hankel matrix used for predictions in (7) will generally be full rank. Hence, the Hankel matrix constraint will imply that any trajectory is possible leading to poor closed‐loop performance of the DeePC algorithm. Furthermore, the online measurements $y_{\text{ini}}$ used to set the initial condition from which the predicted trajectory departs are corrupted by measurement noise, and thus may cause poor predictions. Including a 2‐norm penalty on the difference between the estimated initial condition $Y_{p}g$ and the measured initial condition $y_{\text{ini}}$ coincides roughly with a least‐square estimate of the true initial condition.

Regularization has been proposed as one method to deal with these difficulties and extend the DeePC algorithm to nonlinear noisy systems.^15^ We present a variation of these regularizations in the following *regularized DeePC* optimization problem

(8) $$\begin{array}{ll} \underset{u,y,g}{\text{minimize}}\; & \overset{T_{f}-1}{\underset{i=0}{\sum }}\;c(y(t+i),u(t+i))+\lambda_{s}\|Y_{p}g-y_{\text{ini}}{\|}_{2}^{2}+r(g)\\ \text{subject to}\; & \left(\begin{array}{c} U_{p}\\ U_{f}\\ Y_{f} \end{array}\right)g=\left(\begin{array}{c} u_{\text{ini}}\\ u\\ y \end{array}\right)\\ & u\in {𝒰}^{T_{f}},\;y\in {𝒴}^{T_{f}}, \end{array}$$

where $\lambda_{s}\geq 0$, and $r:{\mathbb{R}}^{T_{d}-T_{\text{ini}}-T_{f}+1}\to {\mathbb{R}}_{\geq 0}$ is a function used to regularize *g*. In comparison to the original regularized DeePC formulation,^15^ we use abstract stage cost and regularization functions *c* and *r*, respectively. These will be made concrete in Section 3.2. We also use the 2‐norm instead of the 1‐norm to penalize the difference between the estimated initial condition $Y_{p}g$ and the measured initial condition $y_{\text{ini}}$. Algorithm 1 below summarizes the DeePC procedure where (8) is implemented in a receding horizon fashion.

Algorithm 1 **Regularized DeePC**

1

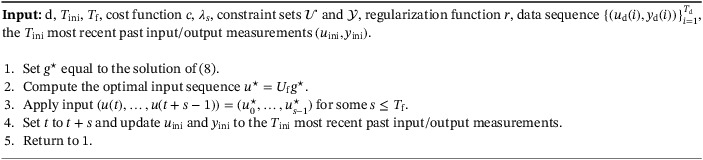

It has been shown that when $r(g)=\lambda_{g}\|g{\|}_{q}$, where $\lambda_{g}\geq 0$ and $q\in {\mathbb{Z}}_{>0}\cup \{+\infty \}$, problem (8) coincides with a distributionally robust problem formulation. Using such a *q*‐norm regularization for the decision variable *g* induces robustness to all systems (nonlinear or stochastic) that could have produced the data in the Hankel matrices (5) within an *s*‐norm induced Wasserstein ball around the data samples used, where $\frac{1}{q}+\frac{1}{s}=1$.^19^, ^27^

The computational complexity of (8) can be characterized by the number of decision variables and constraints. There are $(m+p)T_{f}+\left(T_{d}-T_{\text{ini}}-T_{f}+1\right)$ decision variables, $mT_{\text{ini}}+(m+p)T_{f}$ equality constraints, and $2(m+p)T_{f}$ inequality constraints, when ${𝒰}^{T_{f}}$ and ${𝒴}^{T_{f}}$ are box constraint sets. As is expected of a finite‐horizon optimal control method, the computational complexity grows with the time horizon $T_{f}$. Furthermore, $T_{\text{ini}}$ and $T_{d}$ also affect the computational complexity. The former is related to the observability of the unknown system (2), the latter to the system's dimensionality.

## 3 RESULTS

In this section, we present the results and insights gained by applying DeePC Algorithm 1 described in Section 2.3 for trajectory tracking of the quadcopter system described in Section 2.1. The challenges posed by this application are:

1. The nonlinear and stochastic nature of the quadcopter system requires that the regularization function in (8) and the other hyperparameters offered by the DeePC Algorithm 1 be chosen appropriately for the application at hand. This is addressed by the simulation‐based analysis in Section 3.2.
2. The simulation model is a simplification of the real‐world quadcopter system which neglects complex aerodynamic phenomena, drag, delays in actuation, communication and sensing, and process noise. Essentially, the simulation model contains merely the bare Newtonian dynamics, and even those are subject to parametric uncertainties. Therefore, it is not clear that simulation‐based parameter selection can be directly transferred to real‐world experiments. This is addressed by the experimental results in Section 3.3.

The real‐world results were collected from laboratory experiments conducted using a motion capture system to provide measurements of the position and orientation of the quadcopter at a frequency of 25 Hz. Thus, the sampling time in the discrete‐time dynamics (2) is 40 ms. The laboratory setup was developed as part of a previous work.^35^ To provide the reader with an idea for the scale of the setup, the Crazyflie 2.0^36^ quadcopter weighs 28 grams and a 12 cubic meter flying space was available. Further details on the setup are given in Section 3.3 where the experimental results are presented. The simulation environment uses the model presented in Section 2.1 and the model parameters identified in a previous work.^37^ These model parameters do not match the specific Crazyflie 2.0 used for the experiments, partially due to additional hardware required for detection by the motion capture system.

### 3.1 Data collection

As described in Section 2.3, the input signal used in the Hankel matrices appearing in (7) must be persistently exciting of sufficient order. This data can be collected by injecting a random input sequence, or by performing a manual flight experiment where a human performs the function of the outer controller. For repeatability of results, we chose the former. Two possible choices of random input signals to be applied during the data collection phase are a pseudorandom binary sequence (PRBS) designed for multiple inputs,^38^ or a white noise signal. Both types of perturbations were tested in simulations and showed a negligible difference in the performance of the DeePC algorithm. The results in this paper are presented using a PRBS input signal during the data collection phase because it generally provides better performance for classical system identification techniques.^39^ The input signals applied for data collection consist of the PRBS excitation signal added to an existing controller that maintains the quadcopter around the hover state. The data collected was used to populate the Hankel matrices in (5).

### 3.2 Simulation‐based analysis and insights

The aim of our controller is to track a steady‐state reference $(u_{r},y_{r})\in {\mathbb{R}}^{m}\times {\mathbb{R}}^{p}$. We therefore consider as the cost function *c* the quadratic tracking error between the prediction and the given steady state reference, that is,

(9) $$c(y,u)={\left(y-y_{r}\right)}^{T}Q(y-y_{r})+(u-u_{r})R(u-u_{r}),$$

where Q≽0, R≻0. This cost function is a generalization to the original regularized DeePC^15^ which considers a nonzero steady‐state reference control input $u_{r}$. The values chosen for *Q*, R, and $u_{r}$ are given in Appendix A. The time horizon was chosen as $T_{f}=25$ which corresponds to 1 second in real time. Furthermore, we choose the regularization function in (8) as the following

(10) $$r(g)=\lambda_{g}\;\|g-g_{r}{\|}_{q},\;\text{with}\;g_{r}={\left(\begin{array}{c} U_{p}\\ Y_{p}\\ U_{f}\\ Y_{f} \end{array}\right)}^{†}\left(\begin{array}{c} 1_{T_{\text{ini}}}⊗u_{r}\\ 1_{T_{\text{ini}}}⊗y_{r}\\ 1_{T_{f}}⊗u_{r}\\ 1_{T_{f}}⊗y_{r} \end{array}\right),$$

where $\lambda_{g}\geq 0$, $q\in {\mathbb{Z}}_{>0}\cup \{+\infty \}$, the vector $1_{T_{\text{ini}}}⊗u_{r}$ denotes the stacked column vector consisting of $T_{\text{ini}}$ copies of $u_{r}$ (similarly for $1_{T_{\text{ini}}}⊗y_{r}$), and † denotes the pseudo‐inverse. The vector $g_{r}$ in (10) can be thought of as a “steady‐state trajectory mapper” which linearly combines columns of the Hankel matrix to match the given steady‐state reference trajectory. Among the possibly infinite number of vectors *g* that match the steady state, this is the one with the smallest 2‐norm. In the case when there is no *g* that matches the steady state, $g_{r}$ matches it as closely as possible in the 2‐norm sense. However, this case is unlikely in practice since the Hankel matrix is generally full rank as discussed in Section 2.3. Penalizing the difference between *g* and $g_{r}$ ensures that the stage cost in (8) is zero when the quadcopter is at the steady‐state reference $\left(u_{r},y_{r}\right)$. This is another generalization to the original regularized DeePC,^15^ where only *g* is penalized, and the regularization norm *q* is chosen to be the 1‐norm. We will consider both the 1‐norm and the 2‐norm in this paper.

Under these design choices, the regularized DeePC optimization problem (8) offers several hyperparameters given by:

- $T_{d}$, the total number of data points used to construct the Hankel matrices in (5),
- $T_{\text{ini}}$, the time horizon used for initial condition estimation,
- $\lambda_{s}$, the weight on the softened initial condition constraint,
- $\lambda_{g}$, the weight on the regularization of *g*,
- *q*, the norm used to regularize *g* in (10), and
- *p*, the number of outputs used to construct the Hankel matrices in (5).

Although *p* may seem fixed by the output measurements available, in the case of quadcopter control, it is reasonable to consider whether to use all measurements for position control, that is, set p=5, or use only the position measurements, that is, set p=3.

Note that if one were to approach the control problem through system identification followed by MPC, a number of hyperparameters would also need to be selected. For example, the MATLAB subspace system identification method N4SID requires choosing a model order, weighting scheme, forward estimation and backward prediction horizons, weighting prefilter, output weighting matrix, and other hyperparameters. More generally, system identification for quadcopters requires significant engineering, and previous works resort to the use of partial model knowledge, such as the presence of integrators^40^ or the decoupled nature of the dynamics.^41^, ^42^ This is in addition to the use of full model knowledge in simulating the system and generating the input/output data for identification in these works. Further, the DeePC hyperparameters affect the closed‐loop control performance directly and not through an offline system identification step, which means that they can be easily adapted online on the arrival of new data.

To investigate the effect of the hyperparameters for DeePC, we perform a grid search over the ranges

(11) $$T_{\text{ini}}\in \{1,\ldots ,10\},\;\lambda_{s}\in [10^{5},10^{10}],\;\lambda_{g}\in [10^{0},10^{8}],\;q\in \{1,2\},\;p\in \{3,5\},$$

and a range of $T_{d}$ values that satisfy the minimum data length prescribed by the persistency of excitation requirement from Definition 1. Note that the prediction horizon $T_{f}$, and the cost matrices *Q* and *R* are not parameters unique to the regularized DeePC optimization problem (8), but are also parameters for MPC. For the sake of clarity we do not consider them as hyperparameters in the simulation‐based analysis. Moreover, fixing $T_{f}=25$, and *Q* and *R* as in Appendix A, was sufficient for achieving good closed‐loop performance, and allows for a focus on the other hyperparameters of DeePC. The time horizon used for initial condition estimation $T_{\text{ini}}$, and the number of outputs *p* are also not unique to DeePC, since they are used in some model‐based control approaches which, for example, perform receding horizon state estimation. We consider them as hyperparameters in the simulation‐based analysis in order to gain insights on the implicit state estimation capability of DeePC. For each combination of hyperparameters the following procedure is carried out in simulation. The same procedure is used for the real‐world experiments presented in Section 3.3.

Procedure 1 [Procedure for collecting results in simulation and real‐world experiments] For simulation, the system used was a model of the off‐the‐shelf quadcopter system with dynamics (1) and architecture as in Figure 2, where measurements were affected by zero‐mean Gaussian noise with covariance matrix $\sum_{y}$ as in Appendix A. For the real‐world experiments, the system used was the Crazyflie 2.0.

1. The quadcopter is brought to hover at y=(0,0,1) with a stabilizing controller. The system is excited by adding a PRBS signal to the output of the stabilizing controller, as per Section 3.1, for the input/output data collection step of the DeePC algorithm.
2. The regularized DeePC optimization problem (8) is setup with the input/output data collected in step 1.
3. The DeePC controller is turned on and the quadcopter is commanded to track a diagonal step up from y(0)=(−0.5,−0.5,0.5) to $y_{r}=(0.5,0.5,1.5)$.
4. The resulting closed‐loop tracking error is measured as $\sum_{t=0}^{T_{e}-1}\|y(t)-y_{r}{\|}_{2}^{2}$, where t=0 is the time index at the start of the step trajectory and $T_{e}=250$ is the chosen experiment length, which corresponds to 10 seconds in real time.

#### 3.2.1 Sensitivity to $T_{d}$ and $T_{\text{ini}}$

**FIGURE 3:**
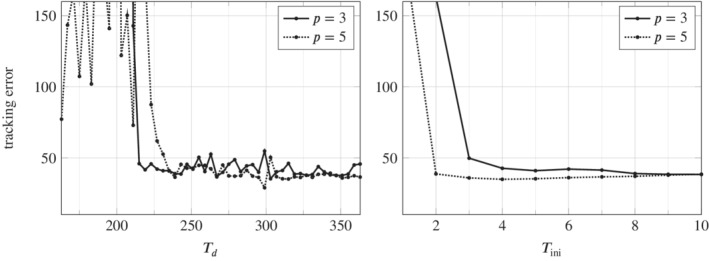
Influence of $T_{d}$ (left) and $T_{\text{ini}}$ (right) on the tracking error. For each point plotted, the tracking error is the minimum achieved over all other hyperparameter combinations considered, with $T_{\text{ini}}=6$ for the left‐hand plot, and $T_{d}=331$ for the right‐hand plot. Evaluating the expression in (12), the Hankel matrix becomes square at $T_{d}=223$ for p=3 and at $T_{d}=287$ for p=5.

As discussed in Section 2.3, for LTI systems the DeePC algorithm requires a minimum number of data points to satisfy the persistency of excitation property. Since we apply the DeePC algorithm to a nonlinear system subject to measurement noise, it becomes unclear as to how many data points are needed in order to construct the Hankel matrices in (5). Figure 3 shows the sensitivity analysis of $T_{d}$ and $T_{\text{ini}}$ on the tracking error. Figure 3 (left) shows the influence of $T_{d}$ on the tracking error, where for each value of $T_{d}$ considered we show the smallest tracking error achieved over all combinations of the other hyperparameters in the grid given by (11) with $T_{\text{ini}}=6$. Similarly, Figure 3 (right) shows the influence of $T_{\text{ini}}$ on the tracking error, where for each value of $T_{\text{ini}}$ considered we show the smallest tracking error achieved over all combinations of the other hyperparameters in the grid given by (11) with $T_{d}=331$.

The key insight from the grid search result in Figure 3 (left) is the distinct improvement in the tracking error of the regularized DeePC algorithm when the number of data points is chosen such that the Hankel matrix appearing in the DeePC optimization problem (8) has at least as many columns as rows. Since the Hankel matrix is generally full rank when the data is obtained from a nonlinear noisy system, having a square Hankel matrix ensures that the subspace spanned by its columns contains the actual subspace of possible trajectories of the system. When the Hankel matrix is slim (i.e., has less columns than rows), this property may not hold; the subspace spanned by the columns of a slim Hankel matrix may not contain the subspace of possible trajectories of the system. This insight is summarized as the following inequality which states that $T_{d}$ should be chosen to be larger than both the minimum amount needed for persistency of excitation in the LTI case and the minimum amount such that the Hankel matrix in (8) is square

(12) $$T_{d}\geq \max\left\{(m+1)(T_{\text{ini}}+T_{f}+n)-1,(m+p+1)(T_{\text{ini}}+T_{f})-1\right\}.$$

Here n=8 is the number of states corresponding to a minimal realization of (1) linearized about hover. Note that the minimum number of data points such that the Hankel matrix in (8) is square is directly affected by the number of outputs *p*. Hence, a larger *p* requires more data points to satisfy the lower bound in (12) and thus results in more decision variables in problem (8). The distinct improvement in the tracking error when $T_{d}$ is chosen such that the Hankel matrix in (8) is square is also observed in a power system application of DeePC.^21^

A similar trend is observed in Figure 3 (right) for $T_{\text{ini}}$ where good tracking performance is achieved for values larger than $T_{\text{ini}}=2$ for p=5, and $T_{\text{ini}}=3$ for p=3. This suggests that more past measurements are needed to estimate the initial condition of the unknown system when p=3. We observed, however, that setting $T_{\text{ini}}=6$ gives steadier flight of the quadcopter. Under noisy measurements, increasing $T_{\text{ini}}$ leads to better initial condition estimates. For the remaining results (simulation and experimental), Procedure 1 was conducted with the number of data points $T_{d}=331$ and with $T_{\text{ini}}=6$. This resulted in good tracking error performance for both p=3 and p=5, while keeping the size of the DeePC optimization problem (8) small enough to be computationally tractable in real‐time.

#### 3.2.2 Sensitivity to $\lambda_{s}$, $\lambda_{g}$, *q*, and *p*

**FIGURE 4:**
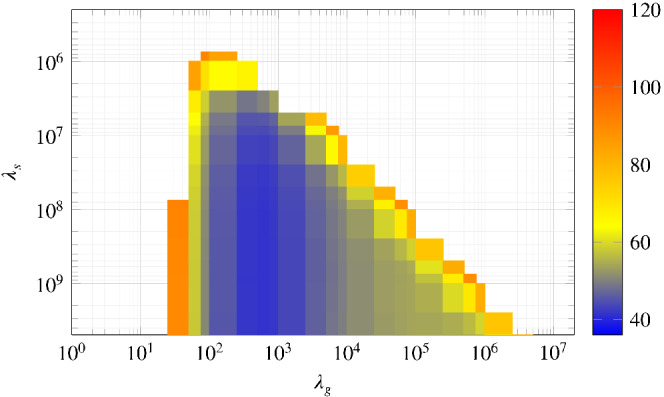
Influence of $\lambda_{g}$ and $\lambda_{s}$ on the tracking error. All other hyperparameters are fixed to the values described in the text. The coloured shading is restricted to the interval (36,120) to sufficiently display the shape of the region shown. The cost increases steeply in regions where the cost is greater than 120, thus the plot is clipped for values greater than 120 for the sake of clarity.

Figure 4 shows the results from the grid search as a heat map over $\left(\lambda_{s},\lambda_{g}\right)$ with fixed values of q=2 and p=3 for the purpose of visualization, and fixed values of $T_{d}=331$ and $T_{\text{ini}}=6$ for the reasons described above. The figure provides the insight that there is a threshold for $\lambda_{s}$ (approximately $\lambda_{s}\geq 10^{7}$) beyond which small tracking error can be achieved. The intuitive explanation for this insight is that a large enough penalization on the softened initial condition constraint ensures that the future predicted trajectory departs from an initial condition close to the actual initial condition. A similar trend of the tracking performance as a function of $\lambda_{s}$ is observed in other numerical case studies of DeePC.^15^, ^21^ This suggests that a tuning guideline for $\lambda_{s}$ is to choose it as large as possible without causing the optimization solver to encounter numerical issues.

**FIGURE 5:**
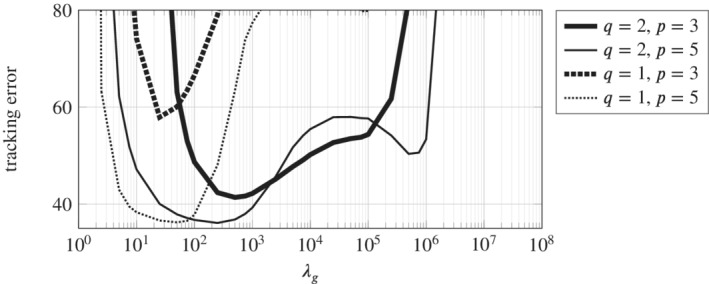
Influence of $\lambda_{g}$, *q*, and *p* on the tracking error with the fixed value of $\lambda_{s}=7.5\times 10^{8}$. Hence for the combination q=2, p=3 (solid thick line) this is the respective slice of Figure 4. The main observation is that the choice q=2, that is, a 2‐norm regularization on decision variable *g*, provides a wider range of $\lambda_{g}$ for which acceptable tracking error is achieved.

Figure 4 also exposes a range for $\lambda_{g}$ in which small tracking error is achieved. To investigate this further we consider the grid search results for all combinations of q∈{1,2} and p∈{3,5}. Figure 5 shows the results from the grid search over $\lambda_{g}$ for a fixed value of $\lambda_{s}=7.5\times 10^{8}$ and for all four combinations of *q* and *p*, for example, the line for q=2, p=3, is the slice of Figure 4 at the fixed value of $\lambda_{s}$. In all cases a small tracking error is achieved for a range of $\lambda_{g}$, although the combination q=1, p=3 performs relatively poorly. This range of $\lambda_{g}$ with acceptable tracking error is wider for q=2 than for q=1, which suggests that for the setup under consideration, 2‐norm regularization is less sensitive to hyperparameter selection than 1‐norm regularization. This observation is supported by observing the heat maps for all four combinations q∈{1,2} and p∈{3,5} as provided in Appendix B. Based on these insights, for the remainder of the results we fix the values $\lambda_{s}=7.5\times 10^{8}$ and q=2 and now investigate in more detail the influence of $\lambda_{g}$ and the choice of output measurements p∈{3,5}.

**FIGURE 6:**
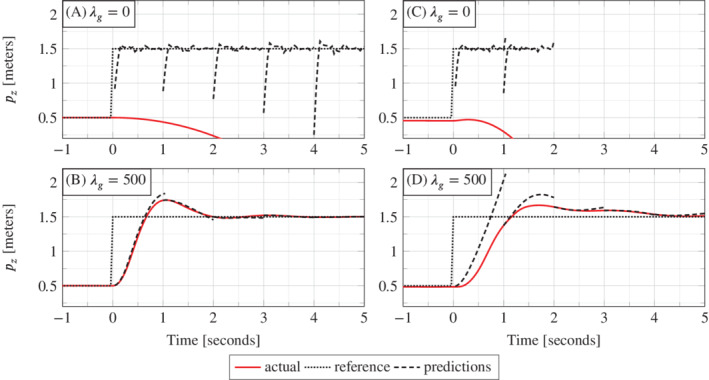
Actual trajectories (solid) versus predicted trajectories from optimization problem (8) (dashed). (A, B) are simulated results and (C, D) are experimental results. The top plots (A, C) are for $\lambda_{g}=0$, and the bottom plots (B, D) are for $\lambda_{g}=500$.

To provide some intuition for how $\lambda_{g}$ influences the optimal solution of the regularized DeePC optimization problem (8) we now take a closer look at the closed loop trajectories resulting from $\lambda_{g}\in \{0,500\}$. Figure 6(A, B) shows the $p_{z}$ coordinate of the simulated closed loop trajectory over time (solid line), the reference $y_{r}$ (dotted line), and the trajectory predicted by problem (8) at representative time instants (dashed line).

In the case of no regularization (Figure 6(A), $\lambda_{g}=0$), the predictions do not correspond to the physics of the model and the actual position diverges, that is, the quadcopter crashes. Since the data used in the Hankel matrix in (8) is obtained from a nonlinear system and is corrupted by measurement noise, then the subspace spanned by the columns of the Hankel matrix is all of ${\mathbb{R}}^{\left(m+p)(T_{\text{ini}}+T_{f}\right)}$. Hence, without regularization on the decision variable *g*, the Hankel matrix predicts that every trajectory is possible. The value $\lambda_{g}=500$ is selected from the grid search result where the DeePC algorithm achieved the smallest tracking error (see Figure 5). We see in Figure 6(B) that desirable reference tracking is achieved and that more physical predictions are computed by the regularized optimization problem (8).

An important distinction between the $\lambda_{g}$ hyperparameter and the $T_{d}$, $T_{\text{ini}}$, and $\lambda_{s}$ hyperparameters discussed above, is that the $\lambda_{g}$ regularization cannot be arbitrarily increased, shown also in Figure 5. The reason is that at a certain level the regularization term r(g) in (8) dominates the tracking error term, leading to poor tracking performance and eventually instability of the system. However, the range of $\lambda_{g}$ resulting in small tracking error is large (e.g., $\lambda_{g}\in [100,10000]$ for q=2, p=3 in Figure 5) indicating robustness to the choice of $\lambda_{g}$.

Hyperparameters $\lambda_{g}$ and *q*, which parameterize the regularization function r(g) in optimization problem (8), are the main parameters of the regularized DeePC algorithm that are not present in model‐based control approaches. These hyperparameters provide distributional robustness against the uncertainty in the system generating the input/output data.^19^, ^27^ Increasing the regularization weight $\lambda_{g}$ provides an increased level of robustness at the cost of being conservative. For intuition, the counterpart of $\lambda_{g}$ in a model‐based approach are model‐order selection parameters that decide how much of the data should be attributed to the model and how much to noise. Similarly, the choice of the regularization norm *q* corresponds to the choice of a loss function in system identification, such as the average or the worst‐case cost. The range of values of $\lambda_{g}$ and *q* which result in a small tracking error depends on the nature of the uncertainty in the system, and the analysis above does not indicate a general guideline that we would expect to apply across multiple systems. Interestingly, however, we observe here and in other applications^20^, ^21^ that the combination $\lambda_{g}=500$ and q=2 performs well. We will explore this empirical observation further in future work.

### 3.3 Real‐world DeePC implementation

We now investigate how the insights gained through the simulation analysis of Section 3.2 transfer to laboratory experiments on a real‐world quadcopter, with the details of the experimental setup provided at the start of Section 3. The experiments are performed as per Procedure 1 (see Section 3.2) and through the results we investigate: (a) whether the insights from the simulation‐based analysis are validated in experiments; (b) whether the hyperparameter values identified from the simulation‐based analysis can be directly transferred to the laboratory environment; and (c) the reliability of the tracking performance achieved.

**FIGURE 7:**
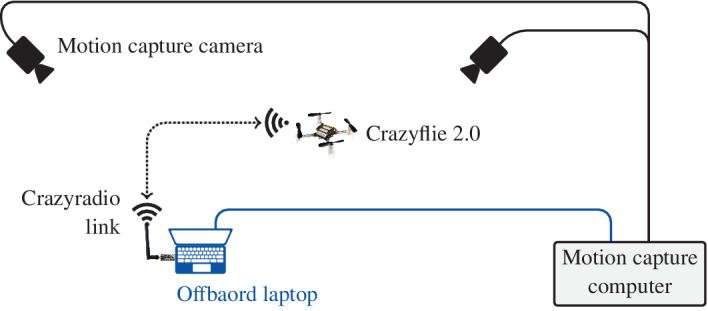
Schematic showing the laboratory setup used to collect the experimental results described in Sections 3.3 and 3.4.

Figure 7 provides a schematic of the laboratory setup used to collect the experimental results. The motion capture system consists of multiple cameras placed around the flying space and connected to a dedicated computer. The software running on the motion capture computer provides accurate measurements^34^ of the position and orientation of the Crazyflie 2.0^36^ quadcopter, i.e., measurements of $\left(\vec{p},\gamma ,\beta \right)$. These measurements are available to an offboard laptop where the outer controller from Figure 2 is implemented. The control decisions of the outer controller, that is $\left(f_{\text{tot}},\omega_{\text{ref},x},\omega_{\text{ref},y}\right)$, are sent via the Crazyradio link to the Crazyflie 2.0 where the firmware provided with the quadcopter runs an onboard controller to track these.

The following analysis of performance on the real‐world system focuses on hyperparameters $\lambda_{g}$ and *p* as these are hyperparameters for which the simulation‐based analysis of Section 3.2 did not provide clear tuning guidelines. On the other hand, the tuning guidelines found in Section 3.2 for hyperparameters $T_{d}$, $T_{\text{ini}}$, and $\lambda_{s}$ generalized well to the real‐world quadcopter, and no significant new insights were observed when varying these hyperparameters in the real‐world. Hyperparameter *q* was set to the 2‐norm because it reduces the number of decision variables in the optimization problem (8) to be solved online and hence reduces the online computation time required. Moreover, the simulation‐based results in Section 3.2 suggest that similarly low tracking error performance is achievable with both q∈{1,2}.

Figure 6(C, D) shows the $p_{z}$ coordinate of the closed loop trajectory, reference, and DeePC predictions when implemented on the quadcopter using the same hyperparameter values as Figure 6(A, B) respectively. The main feature of Figure 6 is that the simulation and experimental results show qualitatively similar closed‐loop trajectories (solid lines) and predictions computed by the DeePC optimization problem (8) (dashed lines). This provides experimental validation of the insight that regularization is required to predict physically reasonable trajectories when applying DeePC to a real system. Moreover, a direct transfer of the hyperparameters selected via simulation to the experiments was possible, and we observed that tracking performance was not significantly improved by adjusting the regularization parameter $\lambda_{g}$. Appendix C provides a similar comparison for hyperparameter values above and below $\lambda_{g}=500$, indicating that the real‐world implementation also achieves the best tracking performance at approximately $\lambda_{g}=500$.

**FIGURE 8:**
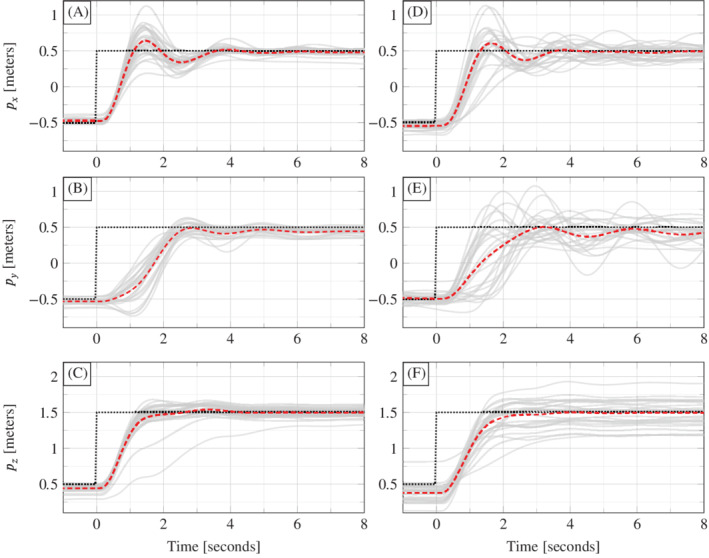
Real‐world quadcopter trajectories (solid grey) for 28 experiments, each with the same change in reference signal (dotted black). Plots (A, B, C) are for p=3 and plots (D, E, F) are for p=5. The dashed lines show the average of the 28 experiments at each time point.

**FIGURE 9:**
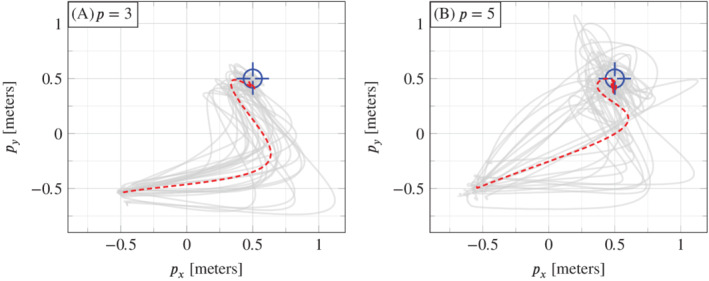
The same data as shown in Figure 8 shown as a top view on the $\left(p_{x},p_{z}\right)$‐plane. Plot (A) is for p=3 and plot (B) is for p=5. The dashed lines show the average at each time point of the 28 real‐world trajectories (solid grey).

To investigate the reliability of the performance observed in Figure 6(D), and also to investigate the influence of hyperparameter *p*, Procedure 1 was repeated in 28 experiments for each of p=3 and p=5. To capture different operating conditions, 14 trials were performed with a fully charged battery and 14 with a partially depleted battery. Figures 8 and 9 and Table 1 summarize the results. Figure 8 shows the position time series data (solid grey) of all 28 trajectories for p=3 (A, B, C) and for p=5 (D, E, F), with the average at each time point (dashed) shown to assist with visualization. Figure 9 shows that same data as a top view.

**TABLE 1:** Real‐world experimental results comparison for p∈{3,5}. Solve time values reported use solver OSQP^43^ on a 64bit Ubunto 16.04 LTS, Intel i7‐8550U, 1.8GHz, 4 Cores, 16GB memory machine.

	Tracking error${}^{a}$			Solve time (ms)		
*p*	Mean	Median	SD	Mean	Median	SD
**3**	75	69	21	4.14	3.92	1.49
**5**	93	86	23	6.66	5.70	4.78

a Computed as described in the Procedure 1.

Quantitatively, Table 1 shows that p=3 achieves a lower tracking error compared to p=5, in terms of mean, median, and SD. This is likely due to the orientation measurements having higher noise than the position measurements. This can be addressed by performing a weighted penalization of $Y_{p}g-y_{\text{ini}}$ using the covariance matrix of the measurement noise. Qualitatively, Figures 8 and 9 suggest that there is less variation in the closed loop trajectories with p=3 than with p=5. This result on the real‐world quadcopter suggests than when applying DeePC to other systems, performance may be improved by discarding measurements with higher noise as long as the system is observable with the remaining measurements.

From the online computation perspective, Table 1 shows that optimization problem (8) is solved sufficiently fast for both p=3 and p=5 considering that output measurements are provided for real‐time implementation at 25 Hz. For the case of p=3, there were 451 optimization decision variables, 168 equality constraints, and 300 inequality constraints. As a point of reference, the optimization problem in the output‐feedback MPC approach of Section 3.4 had 283 optimization decision variables, 208 equality constraints, and the same number of inequality constraints as in the DeePC.

A video of the quadcopter successfully tracking step trajectories and a figure 8 using the DeePC algorithm can be found here: https://doi.org/10.3929/ethz‐b‐000493419.

#### 3.3.1 Summary of hyperparameter selection insights

Through the simulation‐based analysis of Section 3.2 and the real‐world implementation, we gained insights on the selection of the DeePC hyperparameters that we expect to assist with applying the DeePC to other systems. They are summarized as:

- Choose $T_{d}$ as per (12), that is, choose it to be larger than both the minimum amount needed for persistency of excitation in the LTI case and the minimum amount such that the Hankel matrix in (8) is square.
- Choose $T_{\text{ini}}$ by incrementally increasing it until steady tracking is observed. This coincides with a value which both exceeds the lag ℓ of the system in the LTI case and provides good initial condition estimates in the presence of noisy measurements.
- Choose $\lambda_{s}$ as large as possible without causing the optimization solver to encounter numerical issues.
- In regards to *p*, performance may be improved by discarding measurements with higher noise as long as the system is observable with the remaining measurements.

The selection of the regularization function r(g), parameterized in hyperparameters $\lambda_{g}$ and *q*, depends on the nature of the uncertainty in the system generating the input/output data and is expected to vary from one application to another. Preliminary empirical observation suggests that the combination $\lambda_{g}=500$ and q=2 serves as a good initial choice. The 2‐norm regularization is advantageous for real‐time control because it reduces the number of decision variables in the optimization problem (8) to be solved online and hence reduces the online computation time required.

### 3.4 Comparison with model‐based control

The results in Section 3.3 show that DeePC Algorithm 1 achieves good performance for the step reference tracking task specified in Procedure 1 in a data‐driven fashion. We now present a model‐based point of comparison that is developed for linear systems. We take a first‐principles approach that considers the linearization of the quadcopter dynamics (1) about the hover equilibrium point, and we assume that the inner controller tracks the body rates reference signal without dynamics or delays. We use a sampling time of 0.04 seconds, that is, 25 Hz, to convert the continuous‐time linear model to discrete‐time. The resulting linear system model can be readily derived.^44^ Hence we consider a model based‐controller with eight states and three inputs, $\left(\vec{p},\dot{\vec{p}},\gamma ,\beta \right)$ and $\left(f_{\text{tot}},\omega_{\text{ref},x},\omega_{\text{ref},y}\right)$, respectively.

**FIGURE 10:**
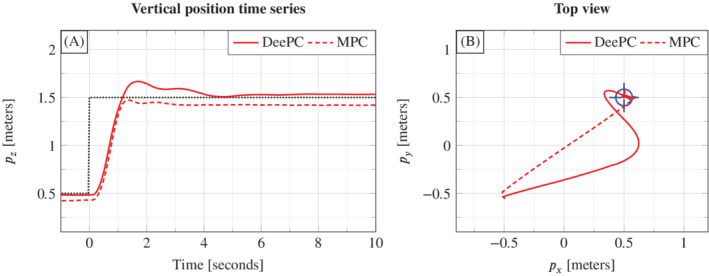
Experimental comparison of data‐enabled predictive control and model predictive control.

The model‐based control method we implement is output‐feedback MPC, as described in Section 2.2. Optimization problem (3) is solved in a receding horizon fashion with the dynamics function *f* replaced by the linear‐time invariant system model described above, the cost function *c* given by (9), and all parameters $\left\{T_{f},Q,R,𝒰,𝒴,u_{r}\right\}$ set to the same values as used for the DeePC as given in Appendix A. The state estimate, $\hat{x}(t)$, is constructed by directly taking the measurements for $\left(\vec{p},\gamma ,\beta \right)$, and $\dot{\vec{p}}$ is estimated as the discrete time derivative of subsequent $\vec{p}$ measurements. Figure 10 compares a trajectory of this first‐principles MPC approach with that of the DeePC. Figure 10(A) shows the time series of the vertical position $p_{z}$, and Figure 10(B) shows the trajectory in the $\left(p_{x},p_{y}\right)$‐plane

Figure 10(A) shows that DeePC and MPC achieve qualitatively similar tracking performance for the vertical position $p_{z}$. Both have a similar rise time and settling time, with the most distinct feature being that the DeePC controller overshoots the reference but then settles to a smaller steady‐state offset. For MPC, this offset is present because there is a model mismatch between the steady‐state input, $u_{r}$, and that needed to maintain the real‐world quadcopter at steady state. As the DeePC controller is provided with the same $u_{r}$, this indicates that the structure of the DeePC controller is able, to some extent, to correct for a mismatch of the steady state input $u_{r}$ provided. Figure 10(B) shows a clear disparity between the tracking performance in the horizontal $\left(p_{x},p_{y}\right)$‐plane. Where the MPC follows an almost straight line trajectory from the starting point to the target, the DeePC controller by contrast has quite different tracking behavior for the $p_{x}$ and $p_{y}$ directions, a trend also observed in Figure 9 and in our simulation‐based tests. This leaves open an interesting direction for further investigation to understand why the DeePC controller produces a faster rise time for the $p_{x}$ direction compared to the $p_{y}$ direction.

Overall, for the quadcopter application we see that DeePC performs similarly to MPC where a first‐principles model is available. This indicates the potential for DeePC to tackle applications where a first‐principles model is either not available or identifying all the necessary model parameters is not conceivable.

#### 3.4.1 Model mismatch

In all of our analysis, the off‐the‐shelf quadcopter is maintained at a zero yaw angle α by the inner controller. At that yaw angle, the quadcopter body frame $x^{\left(B\right)}$, $y^{\left(B\right)}$ axes are aligned with the inertial frame $x^{\left(I\right)}$, $y^{\left(I\right)}$, as is demonstrated in the top view (right) of Figure 1. Therefore, the $x^{\left(I\right)}$ and $y^{\left(I\right)}$ dynamics are decoupled from each other with respect to the body rate reference control inputs of the outer controller $\omega_{\text{ref},y}$ and $\omega_{\text{ref},x}$, respectively. In the real‐world experimental setup, the yaw angle measurement zero reference point must be calibrated by carefully aligning the quadcopter body frame with the inertial frame, and some calibration error is expected. We now consider a case where there is a yaw calibration error of approximately $30^{\circ }$, which is exaggerated for the purpose of demonstration. The quadcopter body frame is rotated by $30^{\circ }$ around the inertial $z^{\left(I\right)}$ axis at the yaw measurement zero reference point, leading to a misalignment in the inertial and body frames that is unknown to the controller.

**FIGURE 11:**
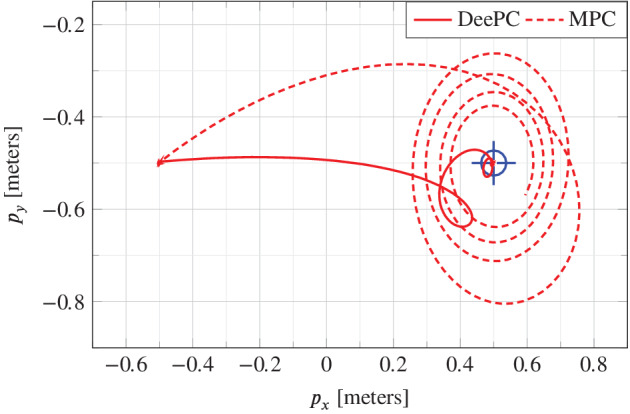
Experimental comparison of data‐enabled predictive control and model predictive control with a yaw angle mismatch.

To capture this yaw miscalibration in simulation, an offset of $30^{\circ }$ between the true yaw angle and the yaw angle measurement available to the controller is induced. Figure 11 shows the simulation results of the quadcopter tracking a 1 meter step in the $p_{x}$ direction with DeePC and output‐feedback MPC. With no knowledge of the coupling between the $p_{x}$ and $p_{y}$ dynamics induced by the misalignment of the inertial and body frames, the output‐feedback MPC controller causes the quadcopter to deviate considerably in the positive $p_{y}$ direction then spiral around the target in the $\left(p_{x},p_{y}\right)$‐plane. By contrast, the quadcopter takes a more direct path to its target under DeePC control. This suggests that the DeePC controller implicitly learns a good mapping between the body rate references $\omega_{\text{ref},x}$, $\omega_{\text{ref},y}$, and the $p_{x}$, $p_{y}$ dynamics from the data collected at the misaligned frames of reference. The mapping is not perfect; a slight spiraling effect as the quadcopter approaches its target is observed, but the improvement to the model‐based approach which equally lacks knowledge of the frames misalignment is apparent.

The yaw angle mismatch is an example of a bias error that can occur when adopting a linear model‐based control approach to a nonlinear system. Such a bias error is present when the linearization is performed at an incorrect operating point. The DeePC algorithm provides some robustness to such a bias error, since it is able to adapt to unknown operating conditions of the system from the data, and also by virtue of the regularization in optimization problem (8). One can further consider a case where the yaw angle measurement calibration drifts slowly over time, and a periodic recalibration is required for a model‐based control approach to perform well. Instead of recalibration, the data in the Hankel matrix in (8) can be updated online in the DeePC approach. We will explore this concept further in future work.

## 4 CONCLUSION

We demonstrated that the regularized DeePC algorithm is suitable for real‐time control of a real‐world quadcopter, thereby bridging the gap between theory and practice. In the process, we performed a sensitivity analysis on the hyperparameters of the DeePC algorithm in simulation, gaining key insights on their effect. These simulation takeaways generalized well to the real‐world quadcopter system, where minimal hyperparameter refining was performed. Through the real‐world implementation, it was demonstrated that the DeePC algorithm is computationally tractable and adequately solvable in real‐time, with solve times far beneath the real‐time requirement. The insights from the simulation and real‐world experiments were condensed into a set of hyperparameter selection guidelines expected to assist with applying the DeePC algorithm to other systems (see Section 3.3.1). Future work includes applying the DeePC algorithm on other real‐worlds systems for which no first‐principles model can be derived.

## CONFLICT OF INTEREST

The authors declare no potential conflict of interest.

## Figures and Tables

**FIGURE B1:**
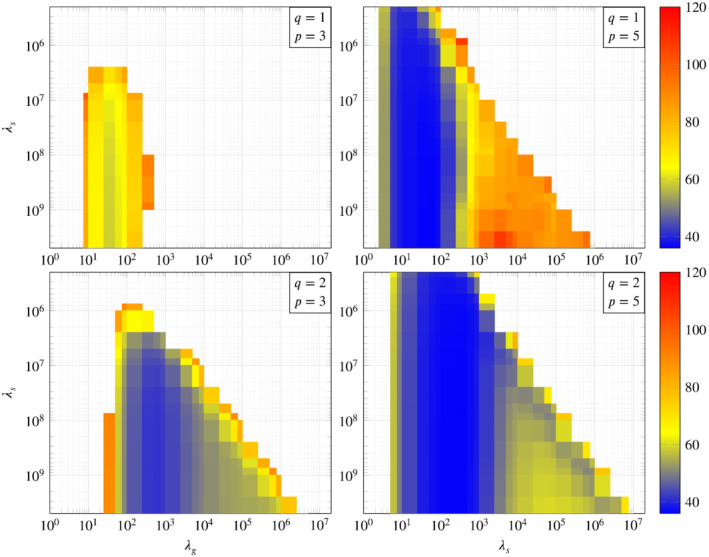
Influence of $\lambda_{g}$ and $\lambda_{s}$ on the tracking error for the four combinations of 1‐norm or 2‐norm regularization (q∈{1,2} respectively) on the decision variable *g*, and p∈{3,5} outputs measured, as labeled on the axes. All other hyperparameters are fixed to the values described in the Section 3.2. The coloured shading is restricted to the interval (36,120) to sufficiently display the shape of each plot. All plots increase steeply for values greater than 120, and the plots are clipped for values greater than 120.

**FIGURE C1:**
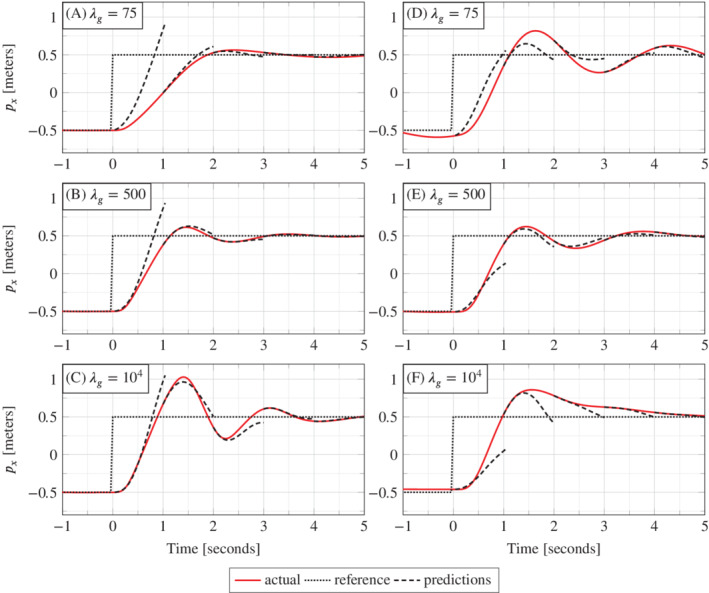
Actual trajectories (solid) versus predicted trajectories (dashed). The plots (A–C) are simulated results and (D–F) are experimental results. To highlight the transferability from simulation to real‐world experiments, for each value of $\lambda_{g}$ (indicated on the plot) all other hyperparameters have the same values. The hyperparameters are selected as those achieving the minimum tracking error in the simulation‐based analysis for the particular value of $\lambda_{g}$.

## Data Availability

The data that support the findings of this study are openly available in “paper‐deepc‐2019‐for‐ijrnl‐data‐gen” at http://doi.org/10.3929/ethz‐b‐000490768, Reference 45.

## Footnotes

* We slightly deviate from the classical definition of a Hankel matrix, which requires it to be square, and allow general dimensions.

## APPENDIX A PARAMETERS FOR IMPLEMENTATION OF THE DEEPC ALGORITHM

### A.1

The following lists the hyperparameters offered by the DeePC algorithm, and the design choices required to specify the quadcopter tracking goal. The value specified in this list is used for all results unless otherwise indicated in the text.

- $T_{d}=331$, the total number of data points used to construct the Hankel matrices in (5),
- $T_{\text{ini}}=6$, the time horizon used for initial condition estimation,
- $\lambda_{s}=7.5\times 10^{8}$, the weight on the softened initial condition constraint,
- $\lambda_{g}=500$, the weight on the regularization of *g*,
- q=2, the norm used to regularize *g* in (10),
- p=3, the number of outputs used to construct the Hankel matrices in (5),
- $T_{f}=25$, the prediction horizon, (corresponds to 1s in continuous time),
- $Q=\left(\begin{array}{ccc} 40 & 0 & 0\\ 0 & 40 & 0\\ 0 & 0 & 40 \end{array}\right)$, the quadratic tracking error cost matrix,
- $R=\left(\begin{array}{ccc} 160 & 0 & 0\\ 0 & 4 & 0\\ 0 & 0 & 4 \end{array}\right)$, the quadratic control effort cost matrix,
- 𝒰, the control inputs constraints set, given by: $f_{\text{tot}}\in [0.1597,0.4791]$, $\omega_{\text{ref},x},\omega_{\text{ref},y}\in \left[-\frac{\pi }{2},\frac{\pi }{2}\right]$,
- 𝒴, the outputs constraints set, given by: $p_{x},p_{y}\in [-4,4]$, $p_{z}\in [0.1,4]$, $\gamma ,\beta \in \left[-\frac{\pi }{6},\frac{\pi }{6}\right]$ when p=5. Note that the constraints on the quadcopter orientation, γ, β, are omitted when p=3,
- $u_{r}=(0.2747,0,0)$, the steady state hovering control inputs,
- $\sum_{y}=\left(\begin{array}{ccccc} 1\times 10^{-8} & 5\times 10^{-9} & 0 & 0 & 0\\ 5\times 10^{-9} & 1\times 10^{-8} & 0 & 0 & 0\\ 0 & 0 & 1\times 10^{-8} & 0 & 0\\ 0 & 0 & 0 & 1.22\times 10^{-5} & 0\\ 0 & 0 & 0 & 0 & 1.22\times 10^{-5} \end{array}\right)$, the covariance matrix of measurement noise in simulation when p=5. Note that when p=3 the covariance matrix is the top left 3×3 block of $\sum_{y}$.

## APPENDIX B FURTHER RESULTS FOR THE GRID SEARCH ANALYSIS

### B.1

For completeness, we include here the results for the grid search analysis, described in Section 3.2, for all hyperparameters considered. Figure B1 bottom left is the same as shown in Section 3.2, and the other plots in Figure B1 are for the remaining combinations of q∈{1,2} and p∈{3,5}.

## APPENDIX C COMPARING SENSITIVITY TO $\lambda_{g}$ IN SIMULATION AND EXPERIMENT

### C.1

Figure C1 shows results similar to Figure 6 for comparing the closed loop trajectories (solid lines) and the predictions computed by the DeePC optimization problem (8) (dashed lines). This shows the same trend that the performance observed in the simulation‐based analysis, Figure C1(A–C), is qualitatively similar to that observed in the real‐world experiments, Figure C1(D–F).

Qualitatively, the best $\lambda_{g}$ chosen in simulation also performs best in reality and results in a similar closed loop trajectory. A smaller value of $\lambda_{g}$ results in a faster but more oscillatory response, and a larger value of $\lambda_{g}$ results in a sluggish response. This figure demonstrates that, despite unmodeled dynamics in simulation, the real‐world system behaves similarly to the simulation model when applying DeePC Algorithm 1. Consequently, simulation‐based hyperparameter selection was adapted on the real system with minimal adjustments required.
